# Guanylate-Binding Protein 1, an Interferon-Induced GTPase, Exerts an Antiviral Activity against Classical Swine Fever Virus Depending on Its GTPase Activity

**DOI:** 10.1128/JVI.02718-15

**Published:** 2016-04-14

**Authors:** Lian-Feng Li, Jiahui Yu, Yongfeng Li, Jinghan Wang, Su Li, Lingkai Zhang, Shui-Li Xia, Qian Yang, Xiao Wang, Shaoxiong Yu, Yuzi Luo, Yuan Sun, Yan Zhu, Muhammad Munir, Hua-Ji Qiu

**Affiliations:** a State Key Laboratory of Veterinary Biotechnology, Harbin Veterinary Research Institute, Chinese Academy of Agricultural Sciences, Harbin, China; b Northeast Agricultural University, Harbin, China; c The Pirbright Institute, Pirbright, Woking, United Kingdom

## Abstract

Many viruses trigger the type I interferon (IFN) pathway upon infection, resulting in the transcription of hundreds of interferon-stimulated genes (ISGs), which define the antiviral state of the host. Classical swine fever virus (CSFV) is the causative agent of classical swine fever (CSF), a highly contagious viral disease endangering the pig industry in many countries. However, anti-CSFV ISGs are poorly documented. Here we screened 20 ISGs that are commonly induced by type I IFNs against CSFV in lentivirus-delivered cell lines, resulting in the identification of guanylate-binding protein 1 (GBP1) as a potent anti-CSFV ISG. We observed that overexpression of GBP1, an IFN-induced GTPase, remarkably suppressed CSFV replication, whereas knockdown of endogenous GBP1 expression by small interfering RNAs significantly promoted CSFV growth. Furthermore, we demonstrated that GBP1 acted mainly on the early phase of CSFV replication and inhibited the translation efficiency of the internal ribosome entry site of CSFV. In addition, we found that GBP1 was upregulated at the transcriptional level in CSFV-infected PK-15 cells and in various organs of CSFV-infected pigs. Coimmunoprecipitation and glutathione *S*-transferase (GST) pulldown assays revealed that GBP1 interacted with the NS5A protein of CSFV, and this interaction was mapped in the N-terminal globular GTPase domain of GBP1. Interestingly, the K51 of GBP1, which is crucial for its GTPase activity, was essential for the inhibition of CSFV replication. We showed further that the NS5A-GBP1 interaction inhibited GTPase activity, which was critical for its antiviral effect. Taking our findings together, GBP1 is an anti-CSFV ISG whose action depends on its GTPase activity.

**IMPORTANCE** Classical swine fever virus (CSFV) is the causative agent of classical swine fever (CSF), an economically important viral disease affecting the pig industry in many countries. To date, only a few host restriction factors against CSFV, including interferon-stimulated genes (ISGs), have been characterized. Using a minilibrary of porcine ISGs, we identify porcine guanylate-binding protein 1 (GBP1) as a potent antiviral ISG against CSFV. We further show that the anti-CSFV action of GBP1 depends on its GTPase activity. The K51 of GBP1, critical for its GTPase activity, is essential for the antiviral action of GBP1 against CSFV replication, and the binding of the NS5A protein to GBP1 antagonizes the GTPase activity and thus the antiviral effect. This study will facilitate the development of anti-CSFV therapeutic agents by targeting host factors and may provide a new strategy for the control of CSF.

## INTRODUCTION

Classical swine fever virus (CSFV) is the causative agent of classical swine fever (CSF), a highly contagious, often fatal viral disease of pigs, leading to significant economic losses in many countries. CSFV is a member of the Pestivirus genus within the Flaviviridae family (1, 2). The virus possesses a single-stranded, positive-sense RNA genome of approximately 12.3 kb (3, 4). Its genome contains a single large open reading frame encoding a precursor polyprotein of 3,898 amino acids (aa) that is co- and posttranslationally processed by viral and cellular proteases, giving rise to four structural proteins (C, E^rns^, E1, and E2) and seven nonstructural proteins (N^pro^, p7, NS2-3, NS4A, NS4B, NS5A, and NS5B) (5, 6).

The innate immune system represents the first line of defense against viral pathogens. Its activation relies on the ability of the specific host pattern recognition receptors (e.g., RIG-I-like receptors, NOD-like receptors, and toll-like receptors) to recognize various components of pathogens (7–9). The signaling pathways are activated by the engagement of these molecules, leading to the production of interferons (IFNs), which bind to their receptors (IFNAR1 and -2), activating the JAK-STAT signal pathway, and transcriptionally induce hundreds of interferon-stimulated genes (ISGs) (10, 11).

The products of ISGs contain numerous antiviral effectors, including the classical ISGs encoding double-stranded RNA-activated protein kinase (PKR), myxovirus resistance protein 1 (Mx1), oligoadenylate synthetase 1 (OAS1) (12), zinc finger protein 313 (ZNF313), interferon-induced protein 44-like (IFI44L), bone marrow stromal cell antigen 2 (BST-2), 2′-5′-oligoadenylate synthase-like protein (OASL), leukocyte surface antigen CD47, and viperin. Viruses in the family Flaviviridae are sensitive to type I IFN both *in vivo* and *in vitro*. IFN treatment induces a large set of ISGs, which protect the host from infection with different viruses. Although hundreds of ISGs have been described, only a few ISGs have been unambiguously identified as having antiviral functions against CSFV.

Recent efforts have focused on screening and identifying antiviral ISGs and deciphering their antiviral mechanisms. IFN effectors differ in the magnitude of their inhibitory activity and present combinatorial antiviral properties. ISGs can target almost any step of the viral life cycle (attachment, entry, uncoating, transcription, genome replication, translation, assembly, or release) (13).

The GTPases are a large family of IFN-induced hydrolases that can hydrolyze GTP. The GTPase family includes four subfamilies: the very large inducible GTPases, the Mx proteins, the immunity-related GTPases (IRGs), and the guanylate-binding proteins (GBPs) (14). These four GTPase subfamilies have several functions, including involvement in the immune responses to viral or bacterial infections (15).

GBPs, with a relative molecular mass of 67 to 69 kDa, consist of an N-terminal globular GTPase domain that binds to GTP and hydrolyzes it into GDP or GMP (16), a C-terminal α-helical regulatory domain, and a short middle domain (17). It has been reported that GBPs are necessary for host mediation of the innate immune responses, which exert antiviral effects against many exogenous pathogens, such as toxoplasmas, chlamydiae, bacteria, and various viruses (18–22).

To date, seven human GBPs (hGBP1 to hGBP7) have been identified, and hGBP1 exhibits antiviral activity against many viruses, including vesicular stomatitis virus (23), encephalomyocarditis virus (23), coxsackievirus (24), hepatitis B virus (24), and hepatitis C virus (HCV) (25). Meanwhile, two porcine GBPs (GBP1 and GBP2) have been reported. It has been demonstrated that GBP1 is upregulated at the transcriptional level in influenza A virus (IAV)-infected pigs (26). However, the potential antiviral activity of GBP1 against other viruses remains elusive.

Here we screened a mini-ISG library against CSFV using lentivirus-delivered cell lines and demonstrated that GBP1 exerts a GTPase-dependent antiviral action against CSFV.

## MATERIALS AND METHODS

### Cells and viruses.

CSFV-permissive PK-15 (porcine kidney) cells were cultured in Dulbecco's modified Eagle's medium (DMEM) (Gibco) supplemented with 5% fetal bovine serum (FBS) (Sigma-Aldrich). Syrian baby hamster kidney (BHK-21) cells and human embryonic kidney (HEK293T) cells were cultured in DMEM supplemented with 10% FBS (free of bovine viral diarrhea virus [BVDV] and anti-BVDV antibodies).

rCSFV-Fluc (27), a reporter CSFV expressing the firefly luciferase (Fluc) gene, was used for screening ISGs. rCSFV-Fluc and the parental virus CSFV strain Shimen were propagated in PK-15 cells. Sendai virus (SeV) was propagated in specific-pathogen-free chick embryos.

### Construction of plasmids and transfection of cells.

Porcine ISGs were cloned into the pFUGW vector (Addgene) to generate pFUGW-ISGs. The porcine GBP1 gene (GenBank accession no. NM_001128473.1) was amplified and cloned into a pCMV-HA empty vector (pHA-EV) (Clontech) or a pCMV-Flag empty vector (pFlag-EV) (Sigma-Aldrich) to generate pHA-GBP1 or pFlag-GBP1, respectively. The CSFV NS5A or NS5B gene was cloned into the pCMV-Myc empty vector (pMyc-EV) (Clontech) to generate pMyc-NS5A or pMyc-NS5B. The primers for the amplification of these genes are listed in Table 1.

**TABLE 1:** Primers used in this study

Primer	Sequence (5′–3′)	Usage
HA-GBP1-F	GCGTCGACCATGGCCTCAAAGGTGCACATG	Amplification of GBP1
HA-GBP1-R	GAAGATCTTTAGCTCAGGAAACATTCTTT	
Flag-GBP1-F	GCCGATATCGATGGCCTCAAAGGTGCACATGC	Amplification of GBP1
Flag-GBP1-R	CGCGGATCCTTAGCTCAGGAAACATTCTTTC	
GST-GBP1-F	CGGGATCCATGGCCTCAAAGGTGCACATGC	Amplification of GBP1
GST-GBP1-R	CCGCTCGAGTTAGCTCAGGAAACATTCTTTC	
Flag-GBP1(R48P)-F	TTGTGGGCCTGTACCCCACAGGCAAATCCTAC	Amplification of GBP1(R48P)
Flag-GBP1(R48P)-R	GTAGGATTTGCCTGTGGGGTACAGGCCCACAA	
Flag-GBP1(K51A)-F	GTACCGCACAGGCGCATCCTACCTGATGAAC	Amplification of GBP1(K51A)
Flag-GBP1(K51A)-R	GTTCATCAGGTAGGATGCGCCTGTGCGGTAC	
Flag-GBP1(1-308)-F	CGGAATTCAATGGCCTCAAAGGTGCACATG	Amplification of GBP1(1-308)
Flag-GBP1(1-308)-R	CGGGATCCTTAGCAGGGCAGGTCCCCAC	
Flag-GBP1(309-591)-F	GCCGATATC GATGGAGAATGCAGTCCTGGC	Amplification of GBP1(309-591)
Flag-GBP1(309-591)-R	CGCGGATCCTTAGCTCAGGAAACATTCTTTC	
Myc-NS5A-F	CCGGAATTCGGATGTCAAGTAATTACATTCTAGAGC	Amplification of NS5A
Myc-NS5A-R	CCGCTCGAGTCACAGTTTCATAGAATACAC	
Myc-NS5A(1-268)-F	CCGGAATTCGGATGTCAAGTAATTACATTCTAGAGC	Amplification of NS5A(1-268)
Myc-NS5A(1-268)-R	CCGCTCGAGTCAAGCAGGCTGCAAGGTTATCTC	
Myc-NS5A(269-497)-F	CCGGAATTCGGATGGTAGTGGTGGATACAAC	Amplification of NS5A(269-497)
Myc-NS5A(269-497)-R	CCGCTCGAGTCACAGTTTCATAGAATACAC	
pFUGW-sfiI(A)-GBP1-F	ACAGGCCATTACGGCCATGGCCTCAAAGGTGCA	Amplification of GBP1
pFUGW-sfiI(B)-GBP1-R	TACGGCCGAGGCGGCCTTATTAGCTCAGGAAACATT	
Q-GBP1-F	GAAGGGTGACAACCAGAACGAC	qRT-PCR for detection of GBP1
Q-GBP1-R	AGGTTCCGACTTTGCCCTGATT	
Q-GAPDH-F	GAAGGTCGGAGTGAACGGATTT	qRT-PCR for detection of GAPDH
Q-GAPDH-R	TGGGTGGAATCATACTGGAACA	

HEK293T or BHK-21 cells were transfected with various plasmids (2 μg each) using 2 μl of X-tremeGENE HP DNA transfection reagent (catalog no. 06366236001; Roche) in 6-well plates (Corning) according to the manufacturer's instructions. At 6 h posttransfection (hpt), fresh DMEM containing 10% FBS replaced the transfection mixture, and the cells were incubated for an additional 48 h.

### Establishment and characterization of cell lines overexpressing ISGs.

To construct stable cell lines overexpressing individual ISGs, HEK293T cells seeded into a 10-cm cell culture dish were transfected with 21 μg of pFUGW-ISGs or pFUGW (serving as a control), together with 14 μg of psPAX2 and 7 μg of pMD2.G. At 6 hpt, the transfection medium was replaced with DMEM supplemented with 10% FBS for 48 h. The recombinant lentiviruses were harvested by collecting the supernatants of the transfected cells. Subsequently, PK-15 cells were transduced with the resulting lentiviruses. The expression of enhanced green fluorescent protein (EGFP)-labeled ISGs or EGFP alone (control) in transduced PK-15 cells (PK-ISG or PK-EGFP cells) was analyzed by Western blotting using a mouse anti-EGFP monoclonal antibody (MAb) (1:1,000) (catalog no. A00185; GenScript).

### Screening of antiviral ISGs.

The stable ISG-overexpressing cell lines (PK-ISG cell lines) seeded into 48-well plates (approximately 2 × 10^5^ cells/well) were cultured with DMEM containing 10% FBS. At 24 h postseeding, cells were infected with rCSFV-Fluc at a multiplicity of infection (MOI) of 0.1 for 48 h and assayed for Fluc activity. The screening was run in triplicate.

The 20 PK-ISG cell lines and the control cell line PK-EGFP cultured in 48-well plates were infected with rCSFV-Fluc for 48 h. The levels of viral replication were expressed as the Fluc activities of the whole-cell lysates.

As controls, PK-15 cells were either left untreated or pretreated with 10, 100, or 1,000 ng of swine IFN-β (catalog no. RP0011S-005; Kingfisher) for 24 h and were then infected with rCSFV-Fluc. Porcine Mx1 (28) was also included as a positive control.

### Luciferase assay.

PK-15 or PK-ISG cells seeded into 24-well plates were infected with rCSFV-Fluc for 48 h. The cells were washed twice with cold phosphate-buffered saline (PBS) and lysed with 100 μl of passive lysis buffer (Promega) in each well, followed by incubation on a shaker for 30 min at 4°C. Then the lysates were centrifuged at 12,000 × *g* for 10 min at 4°C. The supernatants were collected and assayed for Fluc activity using the luciferase reporter assay system (Promega). Luminescence was measured with the TD-20/20 luminometer (Turner Designs) according to the manufacturer's instructions.

### Cell viability assay.

A cell viability assay was performed using the Cell Counting Kit-8 (CCK-8) (catalog no. CK04; Dojindo) according to the manufacturer's instructions.

### RNA interference assay.

Small interfering RNAs (siRNAs) against the porcine GBP1 genes were synthesized by GenePharma. The siRNA sequences targeting GBP1 were CCG AGC UGA CAG AGA GAA UTT (siGBP1-437), GGA GAA CUC ACU CAA GCU UTT (siGBP1-597), and GGA CUC AGA AUU UGU GCA ATT (siGBP1-765). The nontargeting control siRNA (siNC) sequence was UUC UCC GAA CGU GUC ACG UTT. A total of 5 × 10^4^ PK-15 cells were seeded into 24-well plates for 12 h. The cells were transfected with 200 nM siGBP1 or siNC using the X-tremeGENE siRNA transfection reagent (catalog no. 4476093001; Roche) according to the manufacturer's instructions. At 36 hpt, the transfected cells were infected with rCSFV-Fluc or Shimen at an MOI of 0.1. After 2 h, the cells were washed twice with DMEM and incubated at 37°C. At 48 h postinfection (hpi), the cell culture supernatants and cell lysates were harvested for analysis.

### IFA and virus titration.

The titers of CSFV were determined by an indirect immunofluorescence assay (IFA). Briefly, an IFA-based viral titration assay was performed in infected PK-15 cells seeded into 96-well plates (approximately 5 × 10^4^ cells/well) with 10-fold serial dilutions and four replicates for each dilution. After a 48-h incubation, PK-15 cells were washed twice with cold PBS, fixed for 20 min with 4% paraformaldehyde, and permeabilized for 30 min with 0.1% Triton X-100. The fixed cells were incubated with a homemade anti-E2 polyclonal antibody (PAb) (1:100) for 2 h at 37°C, washed five times with PBS, and then incubated with a fluorescein isothiocyanate (FITC)-labeled goat anti-pig IgG (1:100) antibody (catalog no. F1638; Sigma-Aldrich) for 1 h at 37°C. After four washes with PBS, the cells were examined under a fluorescence microscope (TE2000-U; Nikon, Japan) with a video documentation system. Viral titers were calculated by the Reed-Muench method (29) and are expressed as median tissue culture infective doses (TCID_50_) per milliliter.

### qRT-PCR.

Total RNA was extracted from CSFV-infected PK-15 cells or porcine organs using TRIzol reagent (catalog no. 15596026; Invitrogen). The isolated RNA was collected and reverse transcribed into cDNA with avian myeloblastosis virus (AMV) reverse transcriptase XL (catalog no. 2621; TaKaRa) according to the manufacturer's instructions. Genomic RNA copies of CSFV were quantified using a previously described quantitative real-time reverse transcription-PCR (qRT-PCR) assay (30).

### Experimental infection of pigs with CSFV.

To test the expression level of GBP1 in CSFV-infected pigs, various organs (heart, liver, spleen, lung, kidney, and tonsils) were collected from uninfected pigs and from pigs infected with 10^5^ TCID_50_ Shimen at 3 days postinoculation (31). The expression of GBP1 at the transcriptional level was examined by qRT-PCR as described above.

### Dual-luciferase reporter assay system.

In order to use the luciferase reporter assay to analyze the type I IFN pathway, HEK293T cells grown in 24-well plates were transfected with pHA-GBP1 or pHA-EV (0.5 μg each) together with the promoter reporter plasmid pIFN-β-Fluc, pNF-κB-Fluc, or pISRE-Fluc (0.2 μg) and the TK-Renilla luciferase (Rluc) internal reference reporter plasmid (0.01 μg). At 24 hpt, the transfected cells were either stimulated with 20 hemagglutinin units (HAU)/ml SeV or left untreated for another 24 h, and then the reporter activity was analyzed with a TD-20/20 luminometer (Turner Designs). The data were represented as relative expression levels of Fluc and Rluc (Fluc/Rluc ratios).

To determine the effects of GBP1 on the translation efficiency of the CSFV internal ribosome entry site (IRES), HEK293T cells were cotransfected with various plasmids, including different amounts of pFlag-GBP1, 750 ng of pFluc/IRES/Rluc (harboring the Fluc gene under the control of the T7 promoter and the Rluc gene under the control of the CSFV IRES) (32), and 300 ng of pLXSN-T7 (expressing T7 RNA polymerase) (33). At 48 hpt, the reporter activity was measured as described above.

### Co-IP and Western blotting.

For the coimmunoprecipitation (co-IP) assay, HEK293T cells were cotransfected with pFlag-GBP1 and pMyc-NS5A, pMyc-NS5B, or plasmids expressing NS5A mutants (2 μg each). At 48 hpt, the cells were washed twice with cold PBS and lysed with NP-40 lysis buffer containing 1 mM phenylmethanesulfonyl fluoride (PMSF) at 4°C for 1 h. The cell lysates were centrifuged at 12,000 × *g* for 25 min at 4°C, and the supernatants were first precleared with protein G-agarose (catalog no. 11243233001; Roche) and an irrelevant isotype antibody serving as a control at 4°C for 4 h and then incubated with a mouse anti-Flag MAb (catalog no. F1804; Sigma-Aldrich) or a mouse anti-Myc MAb (catalog no. M4439; Sigma-Aldrich) and protein G-agarose at 4°C for 5 h. The agarose was washed five times with NP-40 lysis buffer, and the bound proteins were analyzed by Western blotting.

### GST pulldown assay.

For construction of the pGST-GBP1 plasmid, the GBP1 gene was subcloned into the pGEX-6p-1 expression vector (GE Healthcare). Glutathione *S*-transferase (GST) or the GST-GBP1 fusion protein expressed in Escherichia coli BL21(DE3) cells was purified by glutathione-Sepharose 4B resin (catalog no. 10049253; GE Biosciences) according to the manufacturer's instructions. In brief, expression of GST or GST-GBP1 protein was induced by the addition of isopropylthiogalactoside. The bacterial cells were harvested and resuspended with cold PBS containing 1 mM protease inhibitor PMSF, followed by mild sonication. Subsequently, the soluble GST or GST-GBP1 was incubated with the resin for 5 h at 4°C after centrifugation at 12,000 × *g* for 20 min. The resin was washed five times with cold PBS and incubated for 5 h at 4°C with the lysates of the HEK293T cells transfected with 2 μg of pMyc-NS5A. After five washes with PBS, the bound proteins were analyzed by Western blotting using a rabbit anti-Myc MAb (1:1,000) and a mouse anti-GST PAb (1:2,000) (catalog no. AB101; Tiangen).

### Confocal imaging.

BHK-21 cells grown to 60% confluence were cotransfected with pMyc-NS5A and either pFlag-GBP1 or pFlag-GBP1(K51A) (2 μg each), and PK-15 cells treated with 100 ng of IFN-β were infected with Shimen at an MOI of 0.1 for 48 h. The transfected or infected cells were fixed with 4% paraformaldehyde, blocked with 5% skim milk for 2 h, and then the transfected cells were incubated with a mouse anti-Flag MAb (1:100) or a rabbit anti-Myc PAb (1:100) (catalog no. C3956; Sigma-Aldrich), and the infected cells were incubated with a mouse anti-NS5A PAb (1:100) or a rabbit anti-GBP1 PAb (1:200) (catalog no. ab121039; Abcam) for 1 h. Following 1 h of incubation with an Alexa Fluor 647-conjugated donkey anti-mouse IgG(H+L) antibody (catalog no. 1692912; Life Technologies) and an Alexa Fluor 488-labeled donkey anti-rabbit IgG(H+L) antibody (catalog no. 1674651; Life Technologies), the cells were stained with 4′,6-diamidino-2-phenylindole (DAPI) for 10 min and examined using a Leica SP2 confocal system (Leica Microsystems). The colocalization coefficients were calculated by professional quantitative colocalization analysis software (CoLocalizer Pro, version 2.7.1).

### GTPase assay.

HEK293T cells were transiently transfected with pFlag-GBP1, pFlag-GBP1(R48P), pFlag-GBP1(K51A), or pFlag-EV (2 μg each) and harvested at 36 h. The GTPase activity in cell lysates was determined using an ATPase/GTPase ELIPA (enzyme-linked inorganic phosphate assay) Biochem kit (catalog no. BK051/BK052; Cytoskeleton) that measures the amount of inorganic phosphate (P_i_) generated (absorbance at 360 nm) during hydrolysis on a real-time basis. According to the manufacturer, the absorbance generated in the reaction is directly proportional to the GTPase activity.

To determine the antagonistic effects of the NS5A protein on the GTPase activity of GBP1, HEK293T cells were cotransfected with pFlag-GBP1 and either pMyc-NS5A, pMyc-NS5A(1-268), pMyc-NS5A(269-497), or pMyc-EV (2 μg each) and incubated for 36 h, after which the ELIPA was performed as described above.

### Statistical analysis.

Statistical analysis was performed using SPSS software, version 18.0. Differences between groups were examined for statistical significance using Student's *t* test. An unadjusted *P* value of <0.05 was considered to be significant.

## RESULTS

### Screening of ISGs for the ability to inhibit CSFV replication.

**FIG 1:**
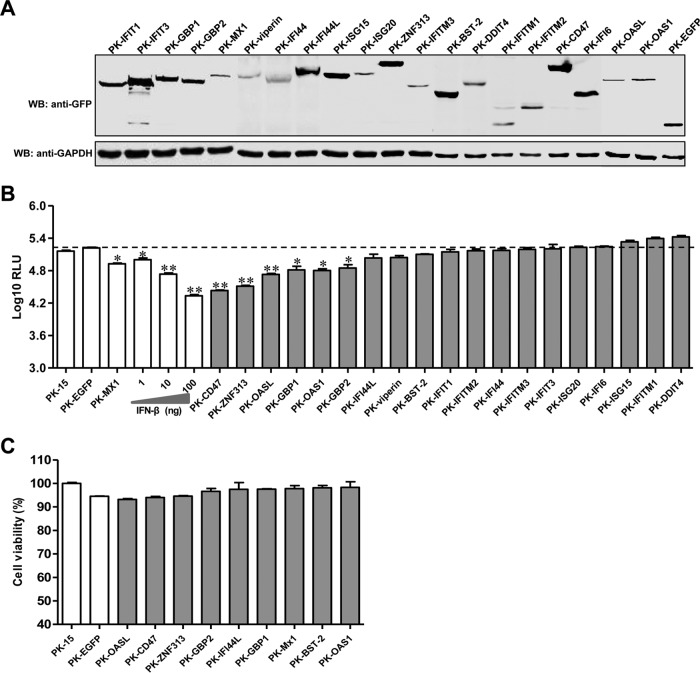
Screening of ISGs for the ability to inhibit CSFV infection. (A) Characterization of ISG expression in PK-ISG cells. Lysates of PK-ISG or PK-EGFP cells were analyzed by Western blotting (WB) using a mouse anti-GFP (1:1,000) or anti-GAPDH (1:1,000) antibody. (B) Effects of ISG expression on rCSFV-Fluc infection. PK-ISG and PK-EGFP cells were seeded into 48-well plates at a density of 2 × 10^5^ per well. At 24 h postseeding, cells were infected with rCSFV-Fluc at a multiplicity of infection of 0.1, cultured for an additional 48 h, and assayed for luciferase activity using the luciferase reporter assay system (Promega). RLU, relative light units. As controls, parental PK-15 cells were either left untreated or pretreated with the indicated concentrations of IFN-β for 24 h, infected with rCSFV-Fluc, and assayed for luciferase activity at 48 h postinfection as described above. An RLU below the dashed line indicates that the candidate is a potential anti-CSFV ISG. Error bars represent standard deviations. Each sample was run in triplicate.*, *P* < 0.05; **, *P* < 0.01. (C) A cell viability assay was performed on cell lines stably overexpressing ISGs.

To screen ISGs for the ability to inhibit CSFV replication, we initially established stable PK-EGFP or PK-ISG cell lines. Twenty ISGs that are commonly induced by IFN-α/β were chosen for screening (Table 2). The expression of the ISGs with the EGFP tag was detected in the cell lines (Fig. 1A).

**TABLE 2:** List of 20 candidate ISGs for establishing stable lentivirus-delivered cell lines

ISG	Length (bp)	GenBank accession no.
IFITM1	375	XM_003124230.2
IFITM2	435	NM_001246214.1
IFITM3	438	NM_001201382.1
IFIT1	1,437	NM_001244363.1
IFIT3	1,530	NM_001204395.1
ZNF313	687	NM_001001869.1
ISG15	504	EU647216.1
ISG20	546	NM_001005351
DDIT4	699	NM_001243452.1
BST-2	534	NM_001161755
MX1	1,992	M65087.1
Viperin	1,089	NM_213817.1
OASL	1,047	NM_001031790
IFI6	393	GACC01000376.1
IFI44L	1,320	XM_003127919.2
IFI44	1,287	XM_005665358.1
GBP1	1,773	NM_001128473
GBP2	1,776	NM_001128474
CD47	912	NM_213982.1
OAS1	1,050	CAA12397.1

As expected, rCSFV-Fluc replication in PK-15 cells was inhibited by both IFN-β and Mx1. Notably, overexpression of GBP1, GBP2, ZNF313, OASL, OAS1, or CD47 significantly reduced Fluc activity in rCSFV-Fluc-infected cells (67.6% ± 3.5% for GBP1, 55.9% ± 15.2% for GBP2, 80.4% ± 1.7% for ZNF313, 59.2% ± 13.9% for OASL, 61.5% ± 6.6% for OAS1, and 83.7% ± 0.9% for CD47), while overexpression of IFI44L, BST-2, or viperin showed modest antiviral activity (31.8% ± 2.4% for IFI44L, 24.3% ± 3.5% for BST-2, and 33.0% ± 1.4% for ZNF313) (Fig. 1B). The antiviral potency of these ISGs was similar to that obtained with IFN-β treatment. The cell viability assay showed that the growth and viability of PK-ISG cells were similar to those of PK-EGFP cells, demonstrating that the effects of these ISGs on the replication of rCSFV-Fluc were not due to cytotoxicity (Fig. 1C).

### GBP1 inhibits CSFV replication.

**FIG 2:**
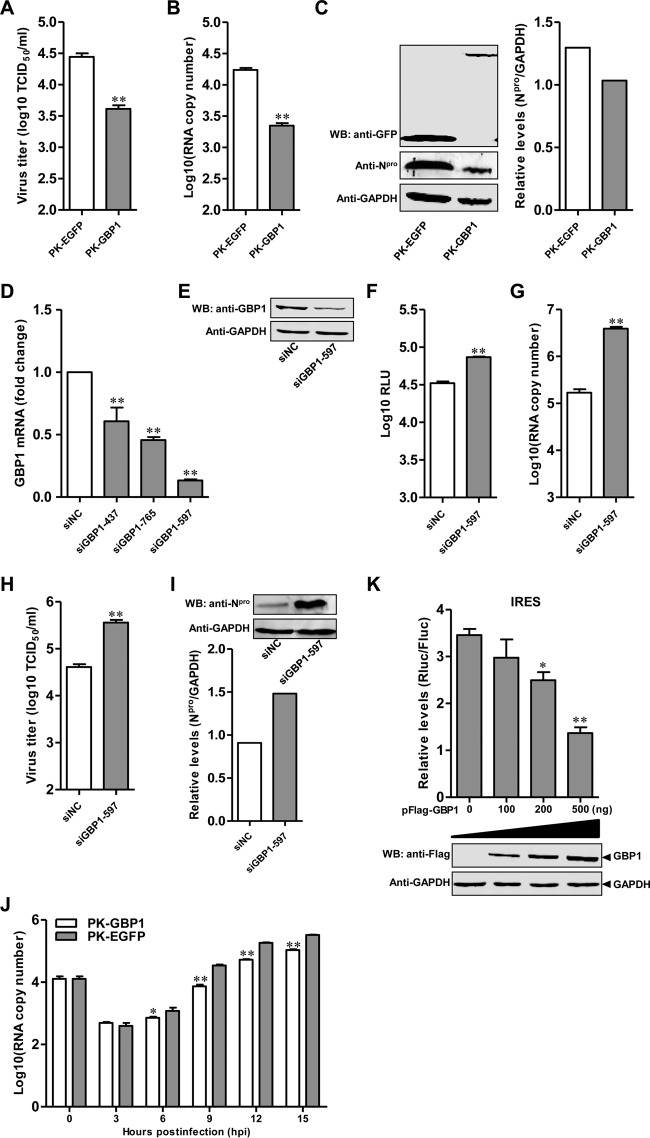
GBP1 inhibits CSFV replication. (A to C) Influence of GBP1 overexpression on Shimen replication. PK-GBP1 and PK-EGFP cells were infected with CSFV strain Shimen at a multiplicity of infection of 0.1 for 48 h. (A) Virus titers in the supernatants were detected by an immunofluorescence assay and are presented as median tissue culture infective doses (TCID_50_) per milliliter. Error bars represent standard deviations. *, *P* < 0.05; **, *P* < 0.01. (B) The genomic copies of CSFV were assessed using a quantitative real-time reverse transcription-PCR assay. (C) (Left) The expression of N^pro^ in cell lysates was analyzed by Western blotting (WB) using a rabbit anti-N^pro^ polyclonal antibody (1:500). GAPDH protein was used as a loading control. (Right) Quantitative analysis of N^pro^ expression in cell lysates was carried out using Odyssey application software, version 3.0. Each sample was run in triplicate. (D and E) Efficiency of knockdown of GBP1 by siRNAs. (D) PK-15 cells transfected with siGBP1 targeting different sequences (siGBP1-437, siGBP1-597, or siGBP1-765) or siNC were harvested at 36 hpt. The efficiency of GBP1 knockdown was checked by qRT-PCR. (E) For Western blotting, PK-15 cells pretreated with 100 ng of IFN-β for 12 h and transfected with siGBP1-597 or siNC were harvested at 36 hpt. GBP1 and GAPDH were detected using a rabbit anti-GBP1 polyclonal antibody (1:500) and a mouse anti-GAPDH monoclonal antibody (1:1,000), respectively. (F) Influence of GBP1 knockdown on rCSFV-Fluc replication. PK-15 cells pretreated with 200 nM siGBP1-597 or siNC for 36 h were infected with rCSFV-Fluc at an MOI of 0.1 for 48 h and assayed for luciferase activity using the luciferase reporter assay system (Promega). RLU, relative light units. (G to I) Effects of knockdown of GBP1 on Shimen replication. PK-15 cells pretreated with 200 nM siGBP1-597 or siNC for 36 h were infected with Shimen at an MOI of 0.1 for 48 h. (G) The number of CSFV genomic copies was assessed using the qRT-PCR assay. (H) The viral titers in supernatants collected at 48 hpi were determined by an immunofluorescence assay and are presented as median tissue culture infective doses per milliliter. (I) The CSFV N^pro^ protein and GAPDH were detected by Western blotting using a rabbit polyclonal anti-N^pro^ antibody (1:500) and a mouse monoclonal anti-GAPDH antibody (1:1,000), respectively. (J) GBP1 targets the early phase of CSFV replication. PK-GBP1 or PK-EGFP cells were infected with Shimen at an MOI of 1. The cells were collected at various time points (0, 3, 6, 9, 12, and 15 hpi). The number of viral genomic copies was determined by qRT-PCR. (K) GBP1 inhibits CSFV IRES activity in a dose-dependent manner. Plasmids pFlag-GBP1 (100, 200, or 500 ng), pFluc/IRES/Rluc (750 ng), and pLXSN-T7 (300 ng) were cotransfected into HEK293T cells. (Top) Luciferase activities were determined and are presented as relative expression levels (Rluc/Fluc). Each sample was run in triplicate. (Bottom) The expression of GBP1 was tested by Western blotting using a mouse anti-Flag MAb (1:1,000).

Now that overexpression of GBP1 was found to inhibit rCSFV-Fluc replication (Fig. 1B), the antiviral activity of GBP1 against CSFV was examined in PK-GBP1 cells. Virus titers in culture supernatants of PK-GBP1 cells were decreased (85.2% ± 3.1%) (Fig. 2A), and the number of viral genomic copies in PK-GBP1 cells was reduced (87.2% ± 2.8%) (Fig. 2B), compared to that in PK-EGFP cells at 48 hpi. Furthermore, the expression level of N^pro^ protein was lower in CSFV-infected PK-GBP1 cells than in CSFV-infected PK-EGFP cells (Fig. 2C).

To examine the effects of GBP1 on CSFV replication, specific siRNAs were used to downregulate GBP1 expression in PK-15 cells, resulting in the efficient decrease of protein expression (Fig. 2D and E). GBP1 expression was knocked down, and the replication of rCSFV-Fluc or Shimen was analyzed. Fluc activity, the number of viral genomic copies, and the virus titer for siGBP1-597-transfected PK-15 cells were significantly increased (2.2-, 18.4-, and 8.8-fold, respectively) over those for siNC-transfected control cells (Fig. 2F to H). Similarly, the expression of the N^pro^ protein in siGBP1-597-transfected cells was increased (Fig. 2I). These results indicate that knockdown of cellular GBP1 enhances CSFV replication, suggesting that GBP1 is a cellular antiviral factor against CSFV infection.

Kinetic studies were performed to investigate the antiviral action of GBP1 on CSFV replication at the transcriptional level. The viral genomic copies in infected cells at various time points postinfection were quantified. The results showed that there were no significant differences in mRNA levels at 3 hpi, demonstrating that GBP1 may not affect the entry of CSFV into PK-GBP1 and PK-EGFP cells. However, the number of viral genomic copies was significantly lower in PK-GBP1 cells than in PK-EGFP cells from 6 to 12 hpi, suggesting that GBP1 targets mainly the early phase of CSFV replication (Fig. 2J).

Furthermore, to determine whether GBP1 affects the translation of CSFV, we tested the effects of GBP1 on CSFV IRES translation efficiency using the dual-luciferase reporter assay. The results showed that GBP1 inhibited CSFV IRES activity in a dose-dependent manner (Fig. 2K).

Taken together, these results show that the antiviral activity of GBP1 acts mainly on the early phase of CSFV replication and inhibits the translation efficiency of the CSFV IRES.

### GBP1 is upregulated upon CSFV infection.

**FIG 3:**
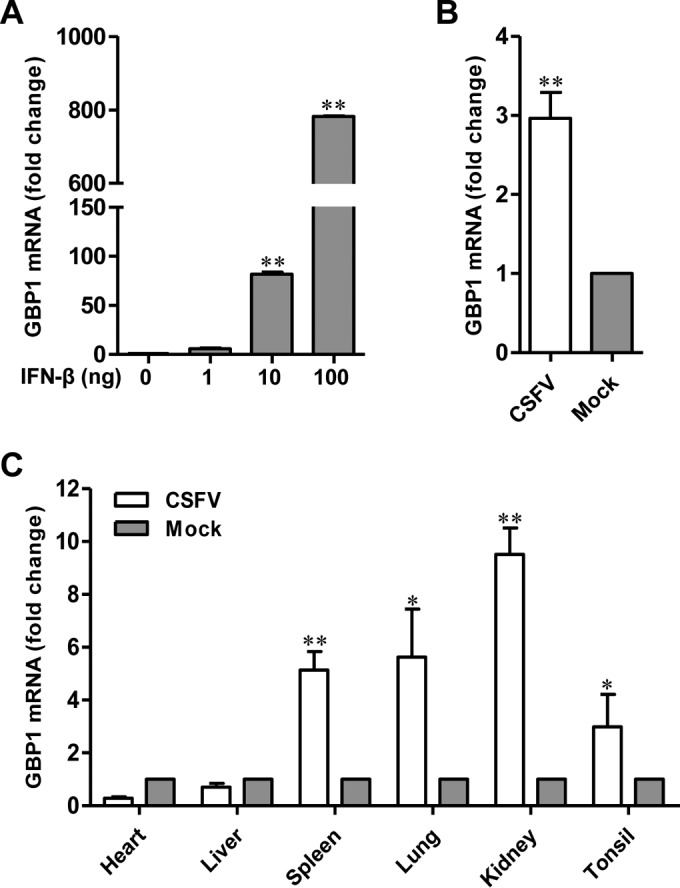
GBP1 is upregulated upon CSFV infection. (A) Expression of GBP1 in PK-15 cells upon IFN-β treatment. GBP1 expression in IFN-β-treated PK-15 cells was examined by qRT-PCR. *, *P* < 0.05; **, *P* < 0.01. (B) Expression of GBP1 in CSFV-infected PK-15 cells. PK-15 cells were infected with CSFV strain Shimen. GBP1 expression was examined by qRT-PCR. Error bars represent standard deviations. (C) Expression of GBP1 in CSFV-infected pigs. Pigs free of CSFV and BVDV were infected with 10^5^ TCID_50_ Shimen. The expression of GBP1 in the hearts, livers, spleens, lungs, kidneys, and tonsils of the infected pigs was examined by qRT-PCR. Each sample was run in triplicate.

To examine the expression of GBP1 following CSFV infection, PK-15 cells were infected with Shimen and examined by qRT-PCR. As controls, PK-15 cells were treated with different amounts of IFN-β. As expected, the expression of GBP1 was upregulated by IFN-β in a dose-dependent manner (Fig. 3A). Similar results were observed in PK-15 cells infected with Shimen (Fig. 3B). We also tested the expression of GBP1 in different organs of pigs infected with Shimen. The results showed that CSFV infection induced GBP1 expression in target organs for CSFV, including the spleen, lung, kidney, and tonsils (Fig. 3C).

### GBP1 does not activate the IFN-β pathway.

**FIG 4:**
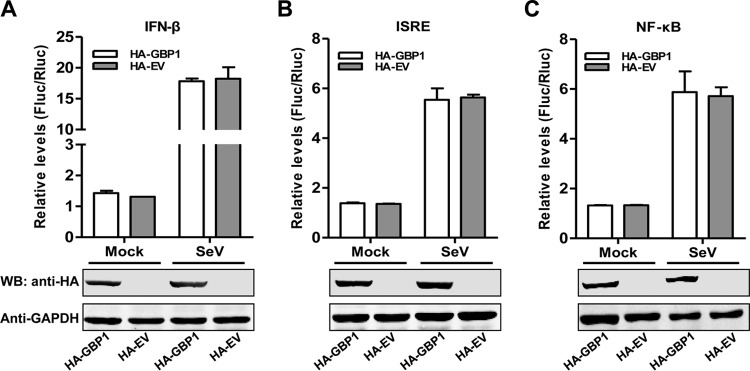
GBP1 does not activate the IFN-β pathway. (Top) HEK293T cells cotransfected with pHA-GBP1 or pCMV-HA (pHA-EV) plus pIFN-β-Fluc and pTK-Rluc (A), pISRE-Fluc and pTK-Rluc (B), or pNF-κB-Fluc and pTK-Rluc (C) for 24 h were either left untreated or treated with 20 HAU/ml SeV for 24 h and were assayed for luciferase activity using the dual-luciferase reporter assay system (Promega). pTK-Rluc was used as an internal reference. Each sample was run in triplicate. Error bars represent standard deviations. HA, hemagglutinin tag. (Bottom) The expression of HA-GBP1 or HA-EV (HA empty vector) in HEK293T cells was determined by Western blotting (WB). GAPDH was used as a loading control.

It has been reported that the expression levels of several ISGs affect the functions of various cellular signaling pathways to exert antiviral activity (25). For instance, GBP1 shows inhibitory effects on dengue virus (DENV) infection by influencing the activity of NF-κB (34). OASL binds directly to RIG-I and positively regulates the expression of IFN-β and ISGs (35). To examine whether GBP1 affects the functions of various cellular signaling pathways, the luciferase activities of lysates from cells transfected with the luciferase reporters driven by IFN-β, interferon-stimulated response element (ISRE), or NF-κB promoters were measured. The results revealed that GBP1 overexpression did not enhance luciferase activities relative to those of empty vector-transfected cells with or without SeV treatment, suggesting that GBP1 does not activate IFN-β, ISRE, or NF-κB promoter activity. The results indicated that GBP1 did not trigger the IFN-β (Fig. 4A), ISRE (Fig. 4B), or NF-κB (Fig. 4C) pathway.

### The K51 of GBP1, which is critical for its GTPase activity, is essential for its inhibition of CSFV replication.

**FIG 5:**
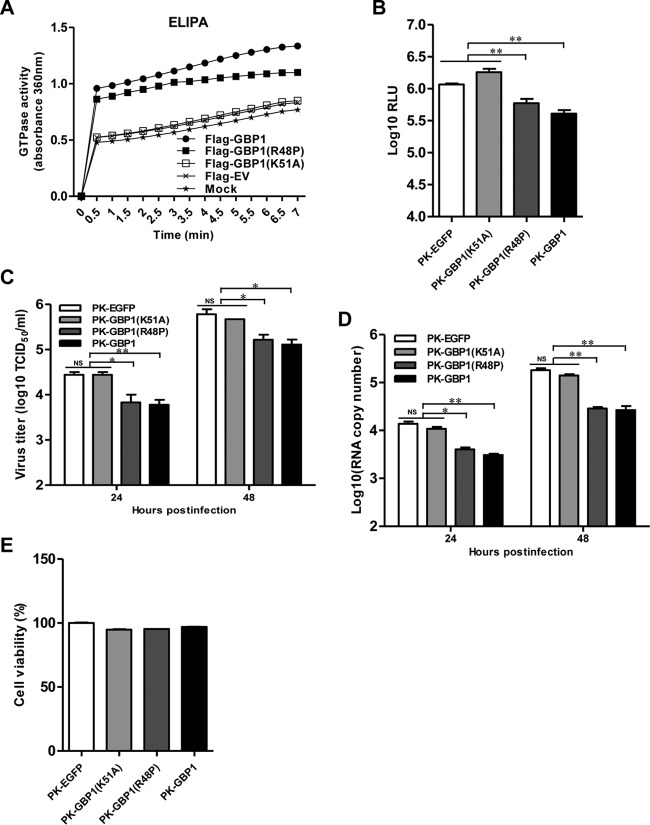
The K51 of GBP1 is required for the inhibition of CSFV replication. (A) HEK293T cells were transfected with pFlag-GBP1, pFlag-GBP1(R48P), pFlag-GBP1(K51A), or pCMV-Flag (Flag-EV) and were harvested at 36 h posttransfection. GTPase activity was measured by an enzyme-linked inorganic phosphate assay (ELIPA). (B) Influence of GBP1(K51A) on rCSFV-Fluc replication. PK-GBP1, PK-GBP1(R48P), PK-GBP1(K51A), and PK-EGFP cells were first infected with rCSFV-Fluc at a multiplicity of infection of 0.1 for 48 h and then assayed for luciferase activity using the dual-luciferase reporter assay system (Promega). Error bars represent standard deviations. NS, not significant; *, *P* < 0.05; **, *P* < 0.01. RLU, relative light units. (C and D) Influence of GBP1(K51A) on CSFV strain Shimen replication. The cell lines were infected with Shimen at an MOI of 0.1 for 48 h. (C) The viral titers in the supernatants collected at 24 and 48 h postinfection were examined by an immunofluorescence assay and are presented as median tissue culture infective doses (TCID_50_) per milliliter. (D) The number of genomic copies of CSFV in PK-GBP1(K51A) cells was determined using a quantitative real-time reverse transcription-PCR assay. Each sample was run in triplicate. (E) Cell viability assay of cell lines stably overexpressing wild-type or mutant GBP1.

It has been reported that the GTPase activity of GBP1 is necessary for its antiviral actions against some viruses, including HCV (20) and IAV (36). Considering that GBP1 exerts an antiviral activity independent of the type I IFN signaling pathway, we hypothesized that its anti-CSFV activity probably depends on its GTPase activity. Since the K51 and R48 of hGBP1 are essential for its GTPase activity (20, 36), we constructed two plasmids expressing two mutants of GBP1, i.e., GBP1(R48P) and GBP1(K51A). To analyze the enzymatic functions of GBP1(K51A) and GBP1(R48P), GTPase activity was examined using ELIPA. GTPase activity was significantly higher in GBP1-expressing cells than in empty vector-transfected cells. The GBP1(K51A) mutant had no GTPase activity, while GBP1(R48P) showed a lower level of GTPase activity than wild-type GBP1 (Fig. 5A). These data suggest that the K51 of GBP1 is crucial for its GTPase activity.

Since K51 is critical for the GTPase activity of GBP1, we further tested if this residue is necessary for the inhibition of CSFV replication. As expected, overexpression of GBP1 significantly reduced the Fluc activity, viral titer, and number of viral genomic copies of CSFV compared with those in the control cells, further confirming the anti-CSFV activity of GBP1. In contrast, the GBP1(K51A) mutant completely lost antiviral activity, while the GBP1(R48P) mutant showed partial antiviral activity, compared with that of wild-type GBP1 (Fig. 5B to D). The cell viability assay showed that those cell lines grew similarly to control cells (Fig. 5E).

The results presented above suggest that the GTPase activity of GBP1 is critical for the inhibition of CSFV replication and that the K51 of GBP1 is essential for its anti-CSFV activity.

### CSFV NS5A interacts with GBP1.

**FIG 6:**
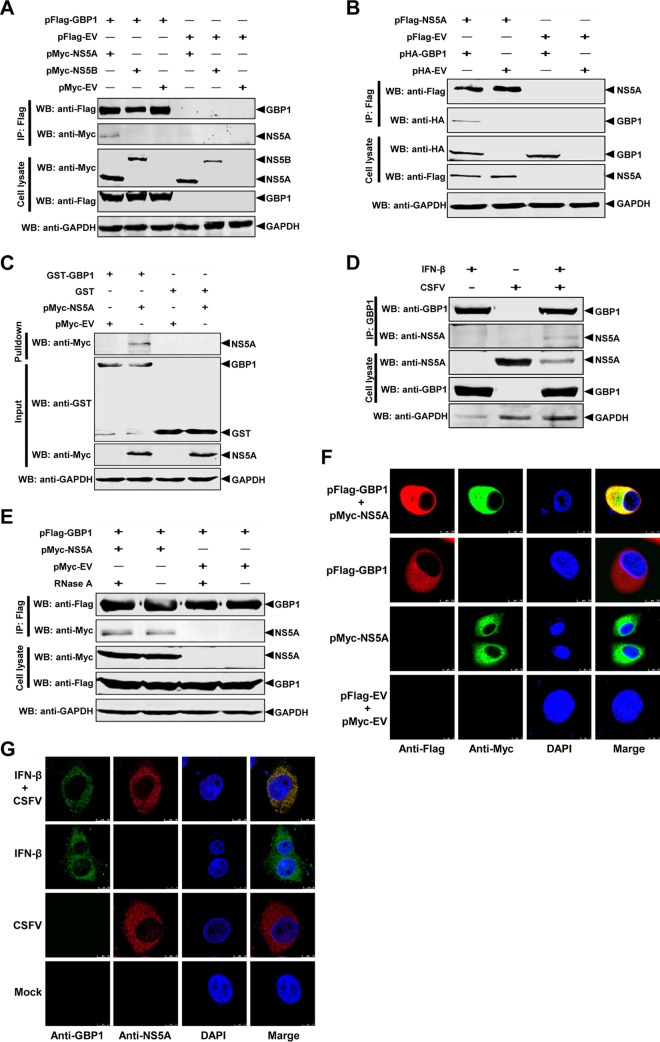
CSFV NS5A interacts with GBP1. (A to D) CSFV NS5A interacts with GBP1. HEK293T cells were cotransfected with pFlag-GBP1 and either pMyc-NS5A, pMyc-NS5B, or pCMV-Myc (pMyc-EV). The cell lysate was harvested. (A and B) Co-IP was performed using an anti-Flag MAb (1:1,000). The precipitated proteins were analyzed by Western blotting (WB) using antibodies against the Myc and Flag tags (A) and the HA and Flag tags (B). (C) For the GST pulldown assay, GST and the GST-GBP1 fusion protein expressed in E. coli BL21 were purified with glutathione resin. The resin was incubated with Myc-NS5A. The bound proteins were determined by Western blotting using a mouse anti-GST PAb (1:2,000) and an anti-Myc MAb (1:1,000). (D) For the endogenous co-IP assay, PK-15 cells were pretreated with IFN-β, infected with CSFV strain Shimen, and subjected to co-IP using an anti-GBP1 MAb (1:1,000). (E) The NS5A-GBP1 interaction is independent of RNA. HEK293T cells were cotransfected with pFlag-GBP1 and pMyc-NS5A. The cell lysate was collected and treated with RNase A. Co-IP was performed using an anti-Flag MAb (1:1,000). (F and G) Colocalization of GBP1 with NS5A. (F) Expression plasmids pFlag-GBP1 and pMyc-NS5A were cotransfected into BHK-21 cells and subjected to a confocal assay. (G) PK-15 cells were pretreated with IFN-β, infected with CSFV strain Shimen, and subjected to a confocal assay. The distribution and colocalization of GBP1 and NS5A were examined using a Leica SP2 confocal system.

It has been reported that various viral nonstructural proteins interact with cellular proteins to evade immune responses. For example, the replicase proteins NS5B of HCV (20) and NS1 of IAV (36) interact with hGBP1. CSFV NS5A and NS5B are main components of the viral replicase complex. Hence, the question of whether CSFV NS5A or NS5B protein interacts with GBP1 to evade its antiviral activity was investigated using co-IP assays. The results showed that Flag-tagged GBP1 interacted with Myc-tagged NS5A but not with Myc-tagged NS5B after incubation with an anti-Flag MAb and protein G-agarose (Fig. 6A). Furthermore, Flag-tagged NS5A was shown to coimmunoprecipitate with hemagglutinin (HA)-tagged GBP1 after incubation with an anti-Flag MAb and protein G-agarose (Fig. 6B). To further confirm the interaction between NS5A and GBP1, endogenous co-IP and GST pulldown assays were performed. The results showed that GST-GBP1 but not GST alone interacted with NS5A (Fig. 6C) and that endogenous GBP1 interacted with CSFV-produced NS5A (Fig. 6D). To preclude nonspecific interaction mediated by RNA, the cell lysates were treated with RNase A prior to co-IP. The co-IP results confirmed that the interaction of GBP1 and NS5A was independent of RNA (Fig. 6E).

We also investigated whether the GBP1 protein colocalizes with NS5A in BHK-21 cells cotransfected with pFlag-GBP1 and pMyc-NS5A. The results showed the colocalization of GBP1 and NS5A in the cytoplasm, with a colocalization coefficient of 0.974 based on the digital analysis of cell images (Fig. 6F). We further examined whether the endogenous GBP1 protein colocalizes with NS5A in CSFV-infected cells. Confocal images showed the colocalization of GBP1 with NS5A in the cytoplasm of CSFV-infected PK-15 cells, with a colocalization coefficient of 0.971 (Fig. 6G).

Taken together, our data demonstrate that GBP1 interacts with the NS5A protein of CSFV.

### GBP1 K51 is critical for the NS5A-GBP1 interaction.

**FIG 7:**
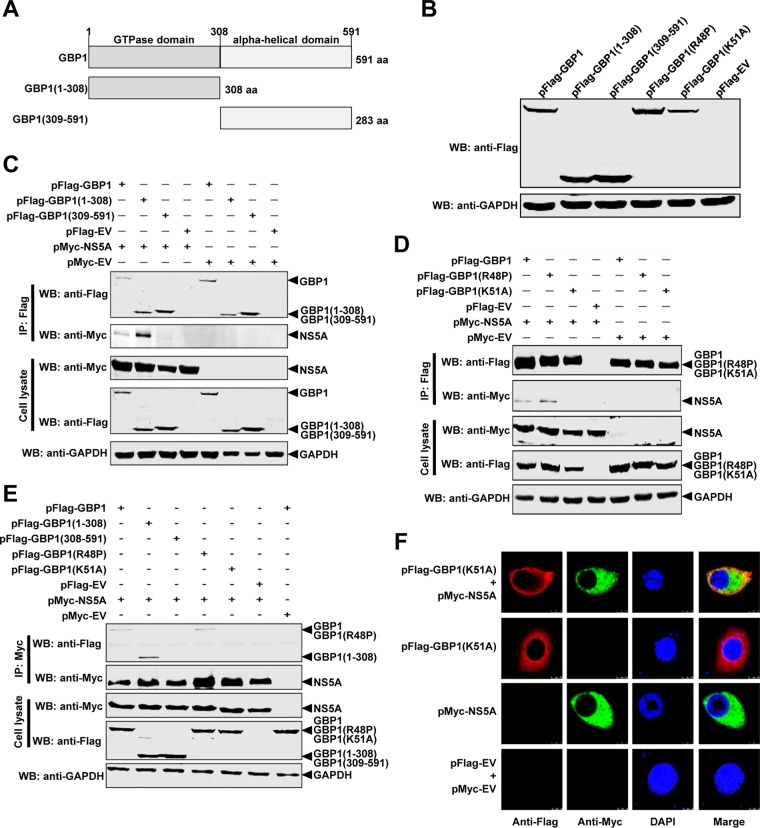
The K51 of GBP1 is critical for the NS5A-GBP1 interaction. (A) Schematic representation of the protein domains of GBP1. The GBP1 protein and two deletion mutants of GBP1 are diagramed. (B) Expression of full-length or truncated forms of GBP1. The indicated expression plasmids were transfected into HEK293T cells, and cell lysates were analyzed by Western blotting (WB) using antibodies against the Flag tag and GAPDH. (C) The N-terminal region of GBP1 is required for its binding to NS5A. HEK293T cells were cotransfected with the indicated expression plasmids expressing Flag-tagged full-length or truncated GBP1 constructs. Cell lysates were collected and subjected to co-IP analysis using an anti-Flag MAb (1:1,000). (D) The K51 of GBP1 is required for binding to NS5A. The interaction of NS5A with Flag-tagged wild-type or mutant GBP1 was examined by co-IP using an anti-Myc MAb (1:1,000). (E) NS5A interacts with GBP1, GBP1(1-308), and GBP1(R48P) but not with GBP1(309-591) or GBP1(K51A). HEK293T cells were cotransfected with the indicated expression plasmids, and co-IP was performed using an anti-Myc MAb (1:1,000). The precipitated proteins were analyzed by Western blotting using antibodies against the Flag and Myc tags. (F) Colocalization of GBP1(K51A) with NS5A. Expression plasmids pFlag-GBP1(K51A) and pMyc-NS5A were cotransfected into BHK-21 cells, and a confocal assay was performed.

To map the region of GBP1 required for binding to NS5A, we constructed two plasmids expressing Flag-tagged truncated mutants of GBP1, i.e., GBP1(1-308) and GBP1(309-591) (20) (Fig. 7A and B). These plasmids were cotransfected with pMyc-NS5A into HEK293T cells and subjected to the co-IP assay. The results showed that NS5A interacted with GBP1 and GBP1(1-308) but not with GBP1(309-591) (Fig. 7C). These findings indicate that the N-terminal globular GTPase domain of GBP1 is critical for the NS5A-GBP1 interaction.

Since the K51 and R48 of hGBP1 are essential for GTPase activity, we determined whether the K51 or R48 of GBP1 was also required for the NS5A-GBP1 interaction. Plasmid pFlag-GBP1(K51A) or pFlag-GBP1(R48P) was cotransfected with pMyc-NS5A into HEK293T cells for the co-IP assay. The results indicated that GBP1 and GBP1(R48P), but not GBP1(K51A), were immunoprecipitated with NS5A (Fig. 7D). The reciprocal co-IP assay also showed that NS5A interacted with GBP1, GBP1(1-308), and GBP1(R48P) but not with GBP1(309-591) or GBP1(K51A) (Fig. 7E).

Furthermore, we also investigated whether the GBP1(K51A) protein colocalizes with NS5A in BHK-21 cells. The results showed that colocalization of GBP1(K51A) and NS5A was not significant (Fig. 7F), with a colocalization coefficient of 0.62. These results suggested that GBP1 K51 is crucial for the NS5A-GBP1 interaction.

### The C-terminal region of NS5A is required for its interaction with GBP1.

**FIG 8:**
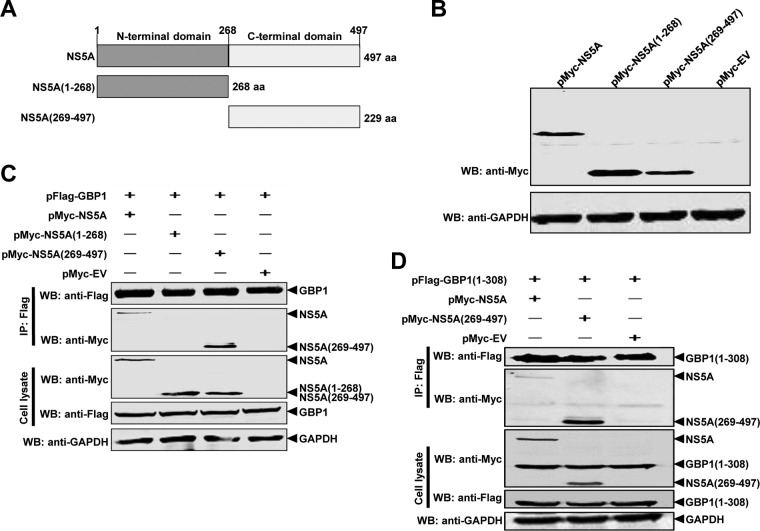
The C-terminal region of CSFV NS5A is critical for the NS5A-GBP1 interaction. (A) Schematic representation of the protein domains of NS5A. Full-length NS5A and two deletion mutants were examined in this study. (B) Expression of full-length and truncated forms of NS5A. The indicated expression plasmids were transfected into HEK293T cells, and cell lysates were analyzed by Western blotting (WB) using antibodies against the Myc tag and GAPDH. (C and D) The C-terminal region of NS5A is required for its binding to GBP1 and GBP1(1-308). The full-length and truncated forms of NS5A were examined by co-IP analysis for interaction with GBP1 (C) or GBP1(1-308) (D). HEK293T cells were transfected with the indicated expression plasmids. Co-IP was performed using an anti-Flag monoclonal antibody (1:1,000). The precipitated proteins were analyzed by Western blotting using antibodies against the Myc and Flag tags.

To further investigate the region of NS5A required for binding to GBP1, we constructed two plasmids expressing Myc-tagged truncated mutants of NS5A (37) (Fig. 8A and B). HEK293T cells were transfected with expression plasmids, and the interaction of GBP1 with NS5A was determined using a co-IP assay. The results revealed that amino acids 269 to 497 at the C-terminal region of NS5A were essential for the interaction with GBP1 (Fig. 8C). As expected, the co-IP results confirmed that NS5A(269-497) coimmunoprecipitated with GBP1(1-308) (Fig. 8D).

### The CSFV NS5A protein antagonizes the antiviral activity of GBP1 by inhibiting its GTPase activity.

**FIG 9:**
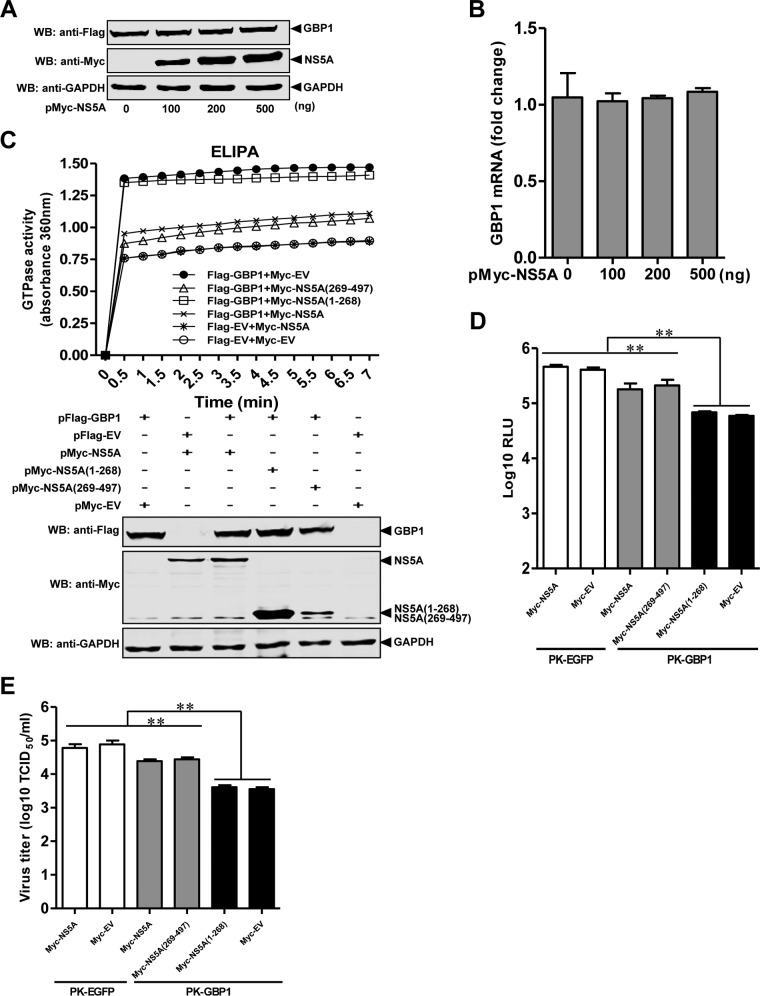
The CSFV NS5A protein antagonizes the antiviral activity of GBP1 by inhibiting its GTPase activity. (A and B) Expression levels of GBP1 in transfected cells. HEK293T cells were transfected with the indicated plasmids and collected at 48 hpt. The expression level of GBP1 was detected by Western blotting (WB) (A) or quantitative real-time reverse transcription-PCR (B). Error bars represent standard deviations. (C) Effects of NS5A on the GTPase activity of GBP1. HEK293T cells were cotransfected with the indicated plasmids and harvested at 36 hpt. GTPase activity was measured by an enzyme-linked inorganic phosphate assay (ELIPA), and the expression of the indicated proteins was verified by Western blotting using antibodies against the Myc tag, the Flag tag, or GAPDH. (D and E) Influence of NS5A overexpression on CSFV in PK-GBP1 cells. PK-GBP1 or PK-EGFP cells were transfected with pMyc-NS5A, pMyc-NS5A(1-268), pMyc-NS5A(269-497), or pCMV-Myc (pMyc-EV). At 24 hpt, the transfected cells were infected with rCSFV-Fluc or CSFV strain Shimen at a multiplicity of infection of 0.1. The cells were collected at 48 h postinfection for analysis of luciferase activity using a luciferase reporter assay system (Promega) (D) and for virus titration using an immunofluorescence assay (E). RLU, relative light units. Each sample was run in triplicate. *, *P* < 0.05; **, *P* < 0.01.

To determine whether NS5A affects GBP1 expression, pFlag-GBP1 and different amounts of pMyc-NS5A were cotransfected into HEK293T cells. The results showed that NS5A did not influence the expression of GBP1 (Fig. 9A and B).

Next, we examined the effects of NS5A on the GTPase activity of GBP1 using ELIPA. The expression of GBP1 significantly increased cellular GTPase activity, while the enhancement of GTPase activity by GBP1 expression was remarkably reduced by NS5A and NS5A(269-497) but not by NS5A(1-268). The data indicated that NS5A interacted with GBP1 and inhibited the GTPase activity of GBP1 (Fig. 9C).

Since NS5A interacts with GBP1 (Fig. 6) and inhibits the GTPase activity of GBP1 (Fig. 9C), we further investigated whether NS5A affects the anti-CSFV activity of GBP1 by conducting a luciferase assay for rCSFV-Fluc and an IFA for Shimen. The results showed that the antiviral effect of GBP1 on CSFV infection was decreased by NS5A and NS5A(269-497) but not by NS5A(1-268) (Fig. 9D and E). These findings suggest that NS5A antagonizes the anti-CSFV activity of GBP1 by inhibiting its GTPase activity.

## DISCUSSION

The persistence of virus replication in host cells is governed by the cellular antiviral system (38) and the ability of the virus to evade or antagonize antiviral responses (20). In this study, we screened ISGs against CSFV and found that GBP1 efficiently suppressed CSFV replication in PK-15 cells. Besides, we demonstrated that GBP1 acted mainly on the early phase of CSFV replication and inhibited the translation efficiency of its IRES (Fig. 2). Furthermore, we found that GBP1 expression was upregulated both *in vitro* and *in vivo* upon CSFV infection (Fig. 3) and that the anti-CSFV effect of GBP1 depends on its GTPase activity but not on the type I IFN or NF-κB pathway (Fig. 4 and 5). Notably, we demonstrated that CSFV NS5A protein interacted with GBP1 and countered the antiviral effect by inhibiting the GTPase activity of GBP1 (Fig. 6 and 9). Collectively, these findings suggest that GBP1 is an anti-CSFV ISG and that this antiviral activity depends on its GTPase activity.

The approaches of overexpression or siRNA-mediated knockdown combined with a reporter virus have been used to screen host genes capable of inhibiting virus infection, such as antiviral ISGs (39–41). Our group has used a reporter virus (rCSFV-Fluc) to screen antiviral siRNAs targeting CSFV proteins (27) or to identify a cellular receptor of CSFV (42). In this study, we preferentially used gene overexpression combined with the reporter virus assay to screen anti-CSFV ISGs, such as GBP1, OASL, and ZNF313 (Fig. 1). Our data suggest that this is a high-throughput, efficient approach.

The GBPs belong to the GTPase family, a group of IFN-induced proteins, which are necessary for host mediation of the immune response to many exogenous pathogens, including chlamydiae, toxoplasmas, bacteria, and various viruses (18–22). Human GBP1 is one of the best-characterized members of the GBPs and has been found to exert antiviral effects against many viruses (23–25). In our study, we demonstrate that porcine GBP1 significantly suppresses CSFV replication in PK-15 cells.

The inhibitory roles and antiviral mechanisms of GBP1 depend on the nature of the pathogen and the infection model. It was speculated that GBP1 might exert anti-cell proliferative activity to restrict the cell-to-cell spread of progeny virus (14, 43). It has been demonstrated that GBP1 inhibits DENV replication by influencing the activity of NF-κB and further contributes to the production of antiviral and proinflammatory cytokines (34). Furthermore, the GTPase domain of GBP1 has been suggested to be critical for both antichlamydial and antiviral effects (21). Other studies have shown that overexpression of GBP1 significantly suppresses HCV (20) or IAV replication (36) through its GTPase activity. In this study, we found that the anti-CSFV effect of GBP1 is independent of the type I IFN and NF-κB pathways (Fig. 4) but dependent on its GTPase activity. This may be one of the mechanisms by which GBP1 exerts its antiviral activity against CSFV.

The HCV NS5B protein interacts with GBP1 and counters its antiviral effects (20). However, in our study, the CSFV NS5A protein interacts with GBP1 and antagonizes the antiviral effect of GBP1 by inhibiting its GTPase activity. CSFV NS5A is a component of the viral replicase complex (44). It has been reported that NS5A can suppress the activity of the IRES located in the 5′ untranslated region (UTR) in the endoplasmic reticulum, induce oxidative stress, interact with the 3′ UTR, and regulate viral replication (45). We demonstrate that GBP1 coordinates with NS5A to reduce CSFV IRES translation efficiency (data not shown). It needs to be further clarified whether the action of GBP1 on the translational activity of the CSFV IRES is associated with its GTPase activity. We also demonstrate that GBP1 acts mainly on the early replication step of the CSFV life cycle.

The highly virulent CSFV strain Shimen could inhibit the IFN-α pathway, causing the loss of endogenous ISGs, such as Mx1 (28). However, our qRT-PCR results show that GBP1 is upregulated at the transcriptional level in PK-15 cells and in pigs after infection with Shimen (Fig. 3), which is consistent with the results of microarray expression profiling (46). GBP1 can be induced by IFN-γ as well as by IFN-α/β, and its induction can be augmented by tumor necrosis factor alpha, interleukin 1, or lipopolysaccharide (47). Thus, we suppose that the upregulated transcriptional level of GBP1 is probably due to the involvement of different host factors.

GBP1 has a functional homology with Mx1 (48); therefore, it is plausible to speculate that porcine GBP1 and Mx1 can synergistically inhibit CSFV replication and may be potential therapeutic agents against CSFV infection. Furthermore, the fusion protein PTD-Mx1 (Mx1 fused with the HIV-1 Tat protein transduction domain [PTD] expressed in E. coli) has been shown to inhibit CSFV replication in a dose-dependent manner (28). Whether PTD-GBP1 also contributes to GBP1-mediated inhibition of CSFV infection requires further investigation.

In conclusion, we demonstrate for the first time that GBP1 is an antiviral ISG against CSFV and acts in the early phase of viral replication in a GTPase activity-dependent manner.
